# An economic analysis of high milk somatic cell counts in dairy cattle in Chiang Mai, Thailand

**DOI:** 10.3389/fvets.2022.958163

**Published:** 2022-11-04

**Authors:** Tosapol Dejyong, Karoon Chanachai, Nathawit Immak, Tippawon Prarakamawongsa, Theera Rukkwamsuk, Damian Tago Pacheco, Waraphon Phimpraphai

**Affiliations:** ^1^Food and Agriculture Organization of the United Nations, Regional Office for Asia and the Pacific, Bangkok, Thailand; ^2^Graduate Student, Bio-Veterinary Science Program (International), Faculty of Veterinary Medicine, Kasetsart University, Bangkok, Thailand; ^3^United States Agency for International Development, Regional Development Mission Asia, Bangkok, Thailand; ^4^The Fifth Regional Livestock Office, Department of Livestock Development, Chiang Mai, Thailand; ^5^Regional Field Epidemiology Training Program for Veterinarians, Department of Livestock Development, Bangkok, Thailand; ^6^Department of Large Animal and Wildlife Clinical Science, Faculty of Veterinary Medicine, Kasetsart University, Nakhon Pathom, Thailand; ^7^Department of Veterinary Public Health, Faculty of Veterinary Medicine, Kasetsart University, Nakhon Pathom, Thailand

**Keywords:** somatic cell count, economic analysis, dairy cattle, investigation, risk mitigation

## Abstract

Mastitis in dairy cattle can lead to significant financial losses due to a reduction in milk yield, the withdrawal period after treatment when milk cannot be sold, and an increase in somatic cell count (SCC) which can reduce the milk's per liter commercial value. Dairy cooperatives point at high-SCC problems as an important factor leading to suboptimal levels of milk quantity and quality. This study aims at describing farm characteristics and milking practices associated with high SCC, identifying risk factors, and assessing the economic loss due to high SCC in three dairy cooperatives in Chiang Mai, Thailand. A cross-sectional study was conducted on 208 dairy cattle farms from July to September 2018. Structured interviews were conducted to collect the data. Univariate and multivariate analyses were performed to determine the degree of association between factors and high SCC. A retrospective cost assessment of high SCC was conducted to estimate the losses in affected farms, and two potential coping strategies were assessed: (1) culling and (2) treating the cow. More than 12% of farms had high SCC (SCC > 500,000 cells/ml). Inappropriate vacuum pressure and inappropriate pulsation rate of milking machines were identified as significant risk factors according to the multiple logistic regression (*P* < 0.01). Both factors can decrease the natural protection of teat tissue, increasing the likelihood of bacterial infection. The average economic loss of high SCC in affected farms was 557 USD for a three-month period. When comparing response strategies (i.e., treatment vs. culling), treating the affected cow was found to be more cost-effective. With a probability of successful treatment of 54%, treating an affected cow leads to 1,158.7 USD in gains over 3 years (vs. doing nothing). The results of this economic analysis can be used to advocate to cooperatives the value of veterinarians and for investigating and treating cases of mastitis.

## Introduction

Poor udder health in dairy cows is an important factor affecting animal welfare, milk production, and milk safety, which ultimately impacts the profitability of farms and dairy cooperatives (1, 2). Mastitis, an inflammation of the mammary gland, is the most common udder health problem. It is characterized by pathological changes in the glandular tissues of the udder, which results in changes in the physical and chemical features of milk (1, 3).

Bovine mastitis is a complex disease associated with various factors, which can be classified as internal, such as the age of the animal, parity, lactation stage, and the animal's health status, and external factors, such as the hygiene of udder management, bedding materials, milking machines, environment management, climate, milking management, and milking practices (3–6). There are two forms of mastitis: clinical mastitis (CM) and subclinical mastitis (SCM). CM involves apparent milk and udder changes upon physical examination including heat, pain, swelling, and redness of the udder, together with reduced milk production and abnormal nature of milk yield (1, 7). SCM is typically associated with no visible changes in the appearance of the milk or the udder, but it can be detected by testing techniques such as milk somatic cell count (SCC) test and pathogen isolation, and it is associated with a 10–20% decrease in milk production (1, 7, 8).

Somatic cells (SCs) consist mostly of milk-secreting epithelial cells (7), with varying levels of blood cells. Normally, the epithelial cells are shed and get renewed. During infection, white blood cells are released as a self-defense mechanism to tackle infection and assist in the repair of damaged tissue. When this happens, increased numbers of epithelial cells are also released into the milk (9). The normal levels of milk SCs or healthy quarters are usually below 100,000 cells/ml; however, they can vary according to lactation cycle (9–11). In Thailand, the normal-SCC level is 400,000–500,000 cells/ml as announced by the Committee of Dairy Cattle Farms and Dairy Products of Thailand (12). High somatic cell counts often related to infections with pathogens such as *Streptococcus uberis, Escherichia coli, Staphylococcus aureus, Streptococcus dysgalactiae*, and *Streptococcus agalactiae* are expected to result in SCC greater than 200,000 cells/ml, whereas minor pathogens such as *Corynebacterium* species and coagulase-negative *Staphylococci* result in SCC in the range of 50,000 to 150,000 cells/ml (9, 11).

Both CM and SCM have a negative impact on the dairy industry and increase veterinary costs (13). CM leads to important losses due to the reduction in milk yields and the withdrawal period after treatment, during which the milk cannot be sold and must be discarded. The California mastitis test (CMT) is commonly used to screen mastitis and decide whether the milk should be discarded. On the contrary, SCM causes a less severe reduction in milk yields and an increase in SCC. Considering that milk with high SCC is categorized as poor-quality milk, its price is penalized. If the SCC is very high, the milk can be rejected by dairy cooperatives, leading to income losses for both the dairy farm and the dairy cooperative (13–18). Therefore, the economic impact of mastitis, either CM or SCM, can be categorized into: (1) milk yield losses, (2) discarded milk because of withholding period from treatment, (3) treatment costs, (4) labor costs, (5) premature culling and replacement, and (6) lethality and the higher risk of co-infection with other diseases (18, 19).

Mastitis can be a common problem, especially in low- and middle-income countries. A meta-analysis found that 16–46% of dairy cows were affected with CM in the studied areas (20). In Thailand, the prevalence of bovine mastitis was 17.92% (CM), with a prevalence of 82.08% (SCM) in Udon Thani province (21) and 40% (SCM) in Khon Kaen province (22).

An effective control program for mastitis at the farm level can be designed regarding identified risk factors from epidemiological studies (23). Potential risk factors for mastitis can be divided into six groups: breed and herd size, housing, hygiene, dairy health, milking management, and milking machine (23). Regarding pulsation vacuum systems of milking machines, an appropriate vacuum pressure is around 40–50 kilopascal (kPa) and a pulsation rate is around 50–60 cycles/minute (24). Research around the world has found similar risk factors for mastitis independently of the local context. A study in Ethiopia revealed that cows at a late lactation stage, milking done by a male worker, and adopting dry cow therapy only at the last milking of lactation, were all determined as risk factors for mastitis (25). While a study in West Shewa zone showed crossbred cattle, old cattle (above 7 years), cows with more than four calves delivered and having a large herd were identified as potential risk factors. In Thailand, there were several studies identifying risk factors for SCM in different provinces. In Khon Kaen, risk factors related to SCM were inappropriate vacuum pressure, cracked teat liners, poor milking management, poor body condition score, and abnormal hoof score (26). In Udon Thani, CM or SCM was strongly associated with irregular post-milking teat dip (27), whereas in Chiang Mai, risk factors were having dairy cows with a history of SCM, having many milking workers, and inappropriate vacuum pressure (28). This study was conducted in 2004 when the Dairy Herd Health Unit was not yet established to support the dairy farms in that area, so risk factors need to be updated.

During an adverse animal health event, stakeholders of the milk value chain in the outbreak area may have different levels of risk perception. This heterogeneity in risk perception combined with different risk profiles can lead to different willingness to adopt risk mitigation measures (29). In general, the level of risk perception depends on the individual judgment about the likelihood that something bad will happen, including losses related to human health, animal health and production, property, reputation, economic, and social wellbeing (29). To mitigate the risk, animal owners are one of the main targets of risk communication campaigns. Communicating the economic impact of diseases during an investigation can improve farmers' awareness, influence their behavior, and ultimately support risk mitigation measures (29–31).

Anecdotal evidence from dairy cooperatives in Chiang Mai, Thailand, points at mastitis as an important factor leading to suboptimal levels of milk quantity and quality in smallholder systems. However, limited epidemiological and economic studies of mastitis (especially SCM) have been conducted, and information regarding management practices and challenges faced by smallholder dairy farmers remains scarce. Thus, efforts to better understand milking hygiene and practices of dairy farmers, mastitis risk factors, and associated economic losses are needed. Such efforts can contribute to the design of appropriate risk reduction measures by the cooperatives and the government, including awareness campaigns. The aim of this study was to identify risk factors for high SCC at the farm level and assess the associated economic loss in three dairy cooperatives in Chiang Mai, Thailand.

## Materials and methods

### Study design and data collection

A cross-sectional study was conducted on all 208 dairy cattle farms of three dairy cooperatives (Mae On, San Kamphaeng, and Phatung cooperatives) located in Mae On and San Kamphaeng districts, Chiang Mai, Thailand, from July to September 2018.

A SCC test was conducted once a month at dairy cooperatives by the Dairy Herd Health Unit of the Fifth Regional Livestock Office. All dairy farms that were cooperative members and had complete SCC results for July, August, and September were included in this study. As announced by the Committee of Dairy Cattle Farms and Dairy Products of Thailand, the normal-SCC level is 400,000–500,000 cells/ml (12). Therefore, dairy farms were identified as high-SCC farms if more than 500,000 cells/ml were found in two out of the three tests.

Face-to-face interviews with farmers from both high- and normal-SCC farms using structural questionnaire were conducted. The investigation team also visited high-SCC farms to observe their practices and conducted checkups on their milking system, as well as provided advice to farmers to reduce the SCC in their milk.

This study was jointly conducted under the agreement of the cooperatives and authorization of the Department of Livestock Development of Thailand. Data collection was designed and conducted through questionnaires, and the existing data from the cooperatives and the government were used in this study as well. No experiments or sampling from animals was conducted in this study. Due to the low risk posed to the participants and animals, the study was conducted under the supervision of local veterinary officers without requiring formal approval from an ethics committee. Before the data collection, the team of researchers and local officers explained the aim of the study to the farmers and provided results and recommendations back to them after the analysis was performed. Confidentiality required the names of farms or farmers to be kept confidential.

### Data analysis

#### Field investigation

The data generated through the interviews were entered into an Excel spreadsheet; data verification and validation were performed. Descriptive statistics of high-SCC farms were computed, including possible causes of high SCC, farm characteristics, farm management, and milking machine and practices. Univariate and multivariate analyses were conducted; odds ratios (OR) and risk ratios (30) with 95% confidence levels and Pearson's Chi-squared tests (or Fisher's exact tests) were computed, and a logistic regression was used to determine the degree of association between factors and high SCC (using Epi Info^TM^, version 7.2.2.6, and R-program, version 3.5.1). All selected risk factors were checked for multicollinearity for categorical variables using the estimation of variance inflation factors (VIFs) (32). Variables with a *p*-value below 0.2 in the univariate analysis were included in the initial multiple logistic regression. Following stepwise backward selection, the variables with a *p*-value below 0.05 were retained in the final model. Furthermore, the overall model fitting of the final model was tested by the Hosmer–Lemeshow goodness-of-fit test (33).

#### Ex post cost assessment of high SCC

According to the standards for raw milk purchases adopted by the Committee of Dairy Cattle Farms and Dairy Products (12), the price that the farm gains depends on multiple factors, such as fat and solid non-fat compositions, number of bacteria, freezing point, as well as SCC, which is influenced by both CM and SCM (Table 1). The cost of high SCC can be calculated by combining the losses due to the reduction in the price of raw milk with the discarded milk that is unsuitable to be sold, which can also contaminate the farm's milk tank. To avoid the contamination of the milk tank, farmers usually screen their milk by using CMT and discharge unsuitable milk. However, as CMT is not a perfect screening test, milk with high SCC could still end up in the tank and lead to price penalties by the cooperative. The calculation is shown as follows (for detailed calculations, refer to Supplementary material):


$$\begin{array}{l} \text{Ex post cost assessment of high SCC}\\ \;=\text{losses due to price reduction of milk due to high SCC}\\ \;+\text{discharged milk.} \end{array}$$

The ex post cost assessment was conducted to determine the economic losses due to high SCC during July–September 2018.

**Table 1:** Price of raw milk according to number of SCC (standards for raw milk purchases announced by the Committee of Dairy Cattle Farms and Dairy Products, 2015).

**Somatic cell count (cells)**	**Price incentive or penalty (THB/kg)**
More than 1,000,000	0.50 reduced
700,001–1,000,000	0.30 reduced
500,001–700,000	0.20 reduced
400,001–500,000	-
300,001–400,000	0.20 added
200,001–300,000	0.30 added
Less than or equal to 200,000	Added

#### Estimation of economic losses of high SCC and cows with mastitis

To identify the optimal response of farmers, the economic losses at the cow level due to severe mastitis were simulated for a three-year period under two different strategies: (1) culling and (2) treatment (using intramammary/systemic antibiotics, non-steroidals (NSAIDs), and vitamin supplements). The three-year period was chosen to consider the medium-term impacts of each strategy. In the case of unsuccessful treatment, we assumed culling as a follow-up action. The exchange rate from August 17, 2018, was used (1 USD = 33.13 THB) for the whole analysis.

Considering that feed costs represent most of the operating costs of smallholder dairy farms, we estimated the baseline profit of dairy farms by subtracting feed costs from the revenue obtained from milk sales. For the culling strategy, the revenue from selling a sick cow is considered. For the treatment strategy, the cost of treatment is added, and the expected profit is estimated in the case of successful and unsuccessful treatment and then aggregated using the likelihood of the treatment being successful. The main parameters for the economic assessment were based on market prices and information provided by experts (veterinary practitioners in dairy cattle in the study area) from the Dairy Herd Health Unit of The Fifth Fifth Regional Livestock Office, Department of Livestock Development of Thailand), and are presented in Table 2. The calculations are described as follows:

**Table 2:** Main parameters of the economic assessment based on market prices (items 1–3) and information (items 4–5) provided by experts from the Dairy Herd Health Unit of The Fifth Regional Livestock Office, Department of Livestock Development of Thailand, at the time of investigation.

**Items**	**Value**
1.Value of a culled cow	453 USD
2.Average treatment cost per cow (including cost of veterinary services, and cost of medicines)	29 USD
3.Daily feed cost in average per a cow	3.3 USD
4.Withdrawal time (including treatment period)	10 days
5.Probability of successful treatment	0.54
6.Discount rate (41)	3%

**Baseline scenario**: Keeping a sick cow untreated and adding its milk to the farm's milk tank (for detailed calculations, refer to equation 3 in Supplementary material).


$$\begin{array}{l} \text{Baseline profit}=\text{NPV}\left(\text{milk revenue}\right)-\text{NPV}\left(\text{feed cost}\right). \end{array}$$

**Strategy A**: Culling a sick cow (for detailed calculations, refer to equation 4 in Supplementary material).

In this case, farmers receive some revenue from selling the sick cow for slaughtering.


$$\begin{array}{l} \text{Profit }=\text{ NPV}(\text{milk}\;\text{revenue})+\text{selling}\;\text{revenue}(\text{sick cow})\\ \;-\text{NPV}(\text{feed cost}). \end{array}$$

**Strategy B.1: Successful treatment** (for detailed calculations, refer to equation 5 in Supplementary material)

A cow with mastitis is treated when farmers can afford the treatment cost and expect that the cow will be fully recovered after the treatment. However, sending the milk to the cooperative during the treatment period is prohibited due to the potential residues of antibiotic and anti-inflammatory drugs.


$$\begin{array}{l} \text{Profit }=\text{ NPV}(\text{milk revenue})-\text{NPV}(\text{feed cost})\\ \;-\text{treatment costs}. \end{array}$$

**Strategy B.2: Unsuccessful treatment followed by culling** (for detailed calculations, refer to equation 6 in Supplementary material).

In this case, after the treatment is unsuccessful, the farmer sells the cow for slaughter, so the milk of the sick cow is never added to the milk tank.


$$\begin{array}{l} \text{Profit }=\text{ NPV}(\text{milk revenue})+\text{selling revenue}(\text{sick cow})\\ \;-\text{NPV}(\text{feed cost})-\text{treatment costs}. \end{array}$$

For the baseline scenario, a cow is kept for 3 years and its milk is not discharged; for the treatment scenario, after a successful treatment period, farmers' income will be based on the milk price and volume associated with a healthy cow (low SCC). Also, water cost, electricity cost, and others are not included in the formula because they are assumed to remain unchanged as the farmer will not reduce the number of workers or other costs just because there is one cow less in the farm.

## Results

### Descriptive statistical information of high SCC, CM, and SCM

**Figure 1:**
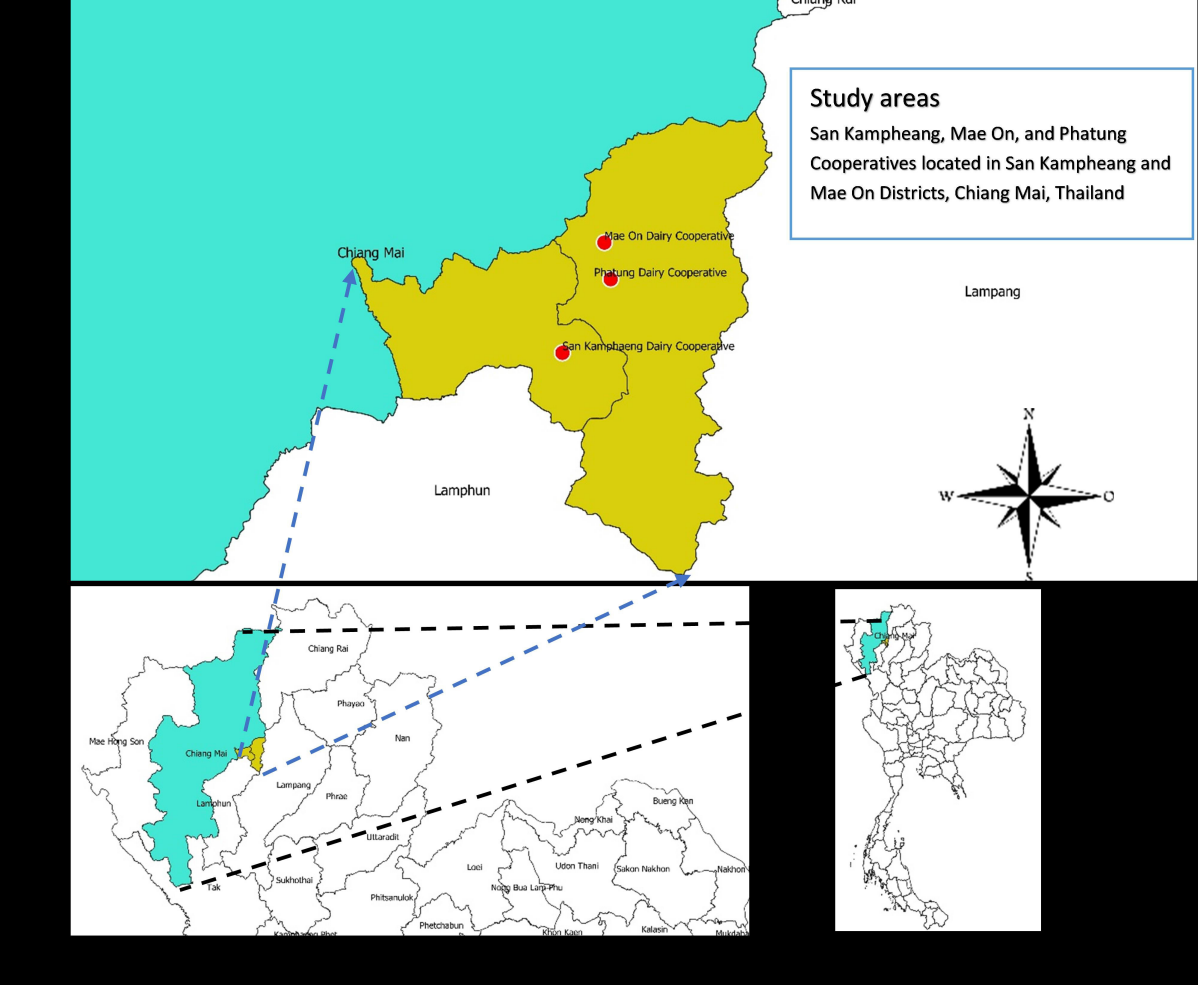
Study areas, San Kamphaeng, Mae On, and Phatung cooperatives located in San Kamphaeng and Mae On districts, Chiang Mai, Thailand. Chiang Mai province is highlighted as the light green area, while the two districts where the three cooperatives are located are highlighted as the yellow area.

There were 208 small dairy farms from the three cooperatives (see Figure 1). Among 208 smallholder farms, the average number of cows and milking cows per farm was 40 and 18 heads, respectively. The average milk sold per day was 198 kg per farm. The average kilogram of milk per milking cow was 11.9 kg. The majority of the farms had tie stall housing with rubber mats as a floor material for the milking area and were not certified as Good Agricultural Practices (GAPs) farms; GAP is a standard for dairy cattle farmers to produce good quality and safe raw milk for consumption (34). Out of the 208 farms, 26 farms (12.5%) were found to be high-SCC farms and at least one cow with CM was found in 128 farms (61.5%) (Table 3).

**Table 3:** Number of categorized farms: high and normal somatic cell count, and farms with CM cow in the three cooperatives.

**Cooperatives**	**High-SCC farms** **(> 500,000 cells/ml)**	**Normal-SCC farms** **(< 500,000 cells/ml)**	**Farms with at least a cow with** **clinical mastitis**	**Total**
San Kamphaeng	8 (13.6%)	51 (86.4%)	30 (50.8%)	59
Mae On	16 (14.2%)	97 (85.8%)	75 (66.4%)	113
Phatung	2 (5.6%)	34 (94.4%)	23 (63.9%)	36
Total	26 (12.5%)	182 (87.5%)	128 (61.5%)	208

Regarding milking practices and management, most of the farms did not clean the milking area daily using disinfectant nor cleaned properly the cows' udders by adopting pre-dipping practice. Most of the farms were engaged in at least one risky practice that can spread mastitis pathogens within the farm, such as using the same towel for cleaning the udders of multiple cows before or after milking. Also, most of the farms did not use CMT for screening individual cows (most of the farms used CMT at bulk milk tank daily).

There were some good milking practices to minimize the risk of mastitis that most of the farms had in place, such as weighting the cluster during milking and keeping cows standing longer after milking. However, regarding the operation of the milking machine management, the majority of farms neither washed the milking cluster before moving to the next cow nor routinely cleaned the equipment of the milking machine (at least once a month).

### Risk factor analysis

As shown in Table 4, the influence of 18 potential risk factors on the incidence of high SCC was analyzed by univariate analysis. This study revealed a significant association of high SCC in farms with respect to five risk factors, with three of them being considered as weakly significant (*p*-value 0.05 to <0.1): (1) average milk sold per day, (2) not cleaning the equipment of the milking machine with chlorinated water, (3) not having pressure gauges, (4) inappropriate vacuum pressure, and (5) inappropriate pulsation rate. With regard to multicollinearity, we found a low correlation among those predictor variables. Applying stepwise backward selection, only two variables remained in the final model: inappropriate vacuum pressure and inappropriate pulsation rate. The final model accounting for both variables achieved a good fit (the Hosmer–Lemeshow test, with a *p*-value closer to (1). Both variables were still significantly different between high-SCC farms and normal-SCC farms from the multiple logistic regression (*p* < 0.01) (Table 5).

**Table 4:** The odds ratio and risk ratio from univariate analysis of high somatic cell count in association with risk factors.

**Variables**	**Odds ratio (95% CI)**	**Risk ratio (95% CI)**	***P*** **-values**
Number of milking cows	-	-	0.13
Average milk sold per day	-	-	0.07
Average discharged milk per day (due to mastitis)	-	-	0.1
No GAP certificates	1.6 (0.69–3.71)	1.23 (0.88–1.72)	0.27
No use of CMT at bulk milk tank (At least once a day)	0.44 (0.09–2.2)	0.53 (0.14–1.98)	0.3
Homemade CMT reagent	1.96 (0.72–5.4)	1.74 (0.79–3.85)	0.18
No cleaning cow's udder before milking	-	-	0.2
No use of a towel per a cow for cleaning cow's udder before milking	0.5 (0.21-1.15)	0.75 (0.5–1.12)	0.99
No use of dry towel after wet towel after milking	1.64 (0.56-4.79)	1.52 (0.63–3.65)	0.36
No use of a dry towel per a cow after wet towel for cleaning cow's udder before milking	2.43 (0.67-8.8)	1.21 (0.97–1.51)	0.16
No use of a dry towel per a cow for cleaning cow's udder after milking	0.70 (0.28–1.77)	0.87 (0.59–1.29)	0.46
No practicing post-dipping	1.43 (0.60–3.36)	1.25 (0.74–2.12)	0.42
No washing milking cluster before milking next cow	1.23 (0.53–2.85)	1.09 (0.78–1.51)	0.63
No cleaning the equipment of the milking machine with chlorinated water	0.46 (0.19–1.12)	0.62 (0.34–1.14)	0.08
Not having pressure gauges	2.13 (0.92–4.97)	1.5 (1.01–2.23)	0.07
Inappropriate vacuum pressure	5.23 (1.99–13.76)	3.21 (1.74–5.91)	< 0.01^*^
Inappropriate pulsation rate	13.27 (3.95–44.59)	5.29 (2.46–11.37)	< 0.01^*^
Having lameness cows	2.08 (0.8–5.44)	1.25 (0.98–1.6)	0.128

* *P*-value < 0.05 is considered as a statistically significant risk factor.

**Table 5:** Association of risk factors with incidence of high somatic cell count from multivariate analysis (multiple logistic regression).

**Variables**	**Coefficients**	***P*** **-Values**
Intercept	−10.7664	< 0.01^*^
Inappropriate vacuum pressure	3.66	< 0.01^*^
Inappropriate pulsation rate	3.00	< 0.01^*^

* *P*-value < 0.05 is considered as a statistically significant risk factor.

### Ex post cost assessment of high SCC (July–September)

The average economic loss associated with high SCC was 533 USD per farm. This represents an aggregated loss of 110,962 USD for all cooperatives, from July to September 2018. Around 10.4% of the total loss was caused by the reduction in raw milk price due to high SCC, while the remaining 89.6% was attributed to discharged milk due to CMT positive.

### Economic gain at the farm level due to severe mastitis for three years under different strategies: Culling or treatment

We considered two main scenarios to manage an individual cow with severe mastitis: culling and treating the cow (followed by culling in case the treatment is not successful). According to the baseline scenario (no action taken), an average farm will experience losses mainly due to the reduction in the milk price due to high SCC. Under this baseline scenario, the farm's profit over a 3-year period was 59,038 USD (Table 6), which is 2,978 USD lower than the profit of a healthy farm.

**Table 6:** Impact of strategies to deal with a cow with clinical mastitis.

**Economic gain strategies**	**Economic gain at the farm level for three years in USD (and THB)**
Healthy scenario (no sick cow)	62,016 USD (*2,051,475* THB)
Keeping a sick cow in a farm (baseline)	59,038 USD (1,955,932 THB)
Healthy scenario–baseline scenario	2,978 USD (98,661 THB)
Economic gain from culling a sick cow	145 USD (4,797 THB)
Economic gain from treating a sick cow	1,186 USD (39,303 THB)

Under the culling strategy, economic gains can be obtained from selling the cow for slaughter and saving on feed costs for one cow over 3 years. Compared with the baseline scenario, the farm will gain 145 USD if the culling strategy is adopted (Table 6). Therefore, culling the cow is a better strategy than keeping the cow untreated.

Under the second scenario, the farm would gain 2,127 USD in the case where treatment is successful (compared with keeping a sick cow in a farm). If unsuccessful, the farm would get some revenue from selling the cow for slaughter, gaining only 82 USD (compared with keeping a sick cow in a farm). Considering that the probability of successful treatment in the studied area was estimated at 54%, the expected economic gain from treating the cow is 1,186 USD (Table 6).

Clinical mastitis (CM) is a common health problem in small dairy farms in Chiang Mai, as shown by the majority of farms having at least one cow with high SCC. However, only a minority of farms had a high-SCC problem. This may be the fact that some farms with SCM remained undetected when using high-SCC information from the cooperatives, as their unqualified milk is usually discarded at the farm (before it reaches the cooperative) by using the CMT to detect individual cows or bulk tanks with high SCC.

Two significant risk factors were identified: inappropriate vacuum pressure and inappropriate pulsation rate of the milking machines. Similar results were obtained by other studies in Khon Kaen, Chiang Mai, and Lamphun, Thailand (26, 28), and Minas Gerais State, Brazil (35). Inappropriate pressure and pulsation rates trigger a decreasing natural protection of teat tissue, which can easily be infected by environmental bacteria. In response to the infection, the inflammatory process starts with the release of the pro-inflammatory cytokine, which leads to a high-SCC problem (36).

Although the association between high SCC and inappropriate milking practices was not significant, there was a higher percentage of malpractices in high-SCC farms compared with normal-SCC farms. Such malpractices have been associated with SCM in previous studies. The malpractices commonly identified included not practicing pre- and post-milking teat disinfection (27, 35, 37) and using a single udder towel for multiple cows (27).

The average (ex post) economic loss due to high SCC between July and September 2018 was 533 USD on average per farm. When aggregating losses for all three cooperatives, the total loss was estimated at 110,962 USD. The calculation included only direct losses measured by the reduction of prices due to high SCC and the discharged milk due to a positive test from the CMT. The ex post calculation did not include other losses associated with CM and SCM, such as milk yield losses along with blind teats and control and prevention costs (14, 15, 18, 19), whereas some costs of normal practices related to mastitis, such as cost of the CMT, cleaning towels, disinfectants, and other general treatments, were observed in all farms (with or without high-SCC problems), so the associated costs do not play a role for the ex post cost assessment. The large economic loss shows that high SCC and mastitis have an impact on dairy farms and cooperatives and could have a detrimental impact on the development of the studied area, with consequences for food safety and security.

According to our analysis, treating the cow is the best strategy as it shows the highest economic gain, when compared with that of culling or doing nothing. When comparing the losses between the farms, those keeping a sick cow within a healthy farm showed a 4.7% loss in revenue. When comparing the culling and treatment strategies with keeping the sick cow on the farm, culling the cow could minimize the loss by about 5%, while treating the cow could minimize the loss by around 41%. Moreover, even with a small probability of successful treatment (above 3.5%), the economic gain for the treatment would be higher than that for culling. Therefore, the farmer should always consider treating the cow as a first option when dealing with mastitis.

One of the limitations in this analysis relates to assuming that after successful treatment, the treated cow goes back to the optimal level of production. In some cases, there can be a long-term reduction in milk production after treatment, due to teat damage, which was not included in the calculation (38, 39). The assumption of culling a cow after an unsuccessful treatment corresponds to the measures recommended by local practitioners and the Dairy Herd Health Unit of the regional livestock office.

When comparing the culling and treating strategies, the economic benefit of treating the cow increases with the time horizon selected for the evaluation, which in this case is 3 years. When expanding the time horizon, the culling strategy could involve replacing the spent cow with a healthy one, which was not included in our analysis. If that is the case, the conclusion could be reverted, depending on relative prices and the timeframe of the evaluation.

Components in the production costs, such as water and electricity costs, were not included as the costs are expected to remain almost the same in smallholder farms, independently of the strategy adopted. While we assumed a fixed likelihood of treatment success, in reality this parameter is influenced by many factors, including individual cow's characteristics (age and number of lactations) and the specific pathogen causing the infection (40).

Major recommendations to farmers and cooperatives were delivered at the end of the study, including adjusting the pulsation and the pressure of the milking system. Awareness campaigns should be considered to promote regular screening of cows in farms with high-SCC problems, along with the treatment of mastitis cows. Moreover, mastitis control and preventive strategies should be designed and implemented by cooperatives, in consultation with government livestock offices. Economic losses due to high SCC, and economic gain from culling and treatment strategies, were communicated to cooperatives and farmers. Considering a lack of veterinarians at the cooperatives who can support disease control and prevention activities, and the large loss of high SCC when aggregating the costs at the cooperative level, the investment of disease mitigation measures should be considered by cooperatives' managers, such as setting up extension teams or hiring/out-sourcing services to improve milk quality at the farm level. When presenting the results of this investigation, including the economic analysis, the awareness among farmers and cooperatives increased. In response, the cooperatives announced the recruitment of more veterinarians, after noting that the losses associated with high SCC exceeded the cost of recruiting more veterinarians to support farmers in managing their milking practices and machines. Also, the Dairy Herd Health Unit of the Fifth Regional Livestock Offices decided to inspect the milking systems and encourage dairy farmers to improve their management practices after the results of this study were presented. Lastly, the methodology of this study which is an integration of disease investigation and economic analysis could be generally utilized to enhance disease mitigation measures among stakeholders. The results from this study such as identified risk factors and economic losses can also be used to improve disease control programs at both sub-national and national levels as well as in other counties where a dairy cattle industry is similar to Thailand.

In the current study, high-SCC problems were notified by the cooperatives, so we may be underestimating the actual number of farms with high-SCC problems, as some of them may practice milk disposal before the delivery of milk to cooperatives based on positive CMT (high SCC) at bulk milk tanks. The SCC test at the cooperatives was conducted on a monthly basis, and it may not be sufficient to detect the problem that is normally based on daily practice and management. Hence, future studies can generate more accurate results if the CMT scores of individual cows are recorded and used to improve the case definition.

## Data availability statement

The raw data supporting the conclusions of this article will be made available by the authors, without undue reservation.

## Ethics statement

Ethical review and approval was not required for the animal study because this study was jointly conducted under the agreement of the cooperatives and authorization of Department of Livestock Development of Thailand. Data collection was designed and conducted through questionnaires and existing data from cooperatives and the government. No experiments or samples from animals were done and collected under this field study. Due to nature of the study of the outbreak investigation and the low risk posed to the participants and animals, the study was conducted under the supervision of local veterinary officers' without requiring formal approval from an Ethics Committee.

## Author contributions

TD, KC, TP, DT, and WP contributed to initiating the topic, the objective, and the methodology of the study. TD, KC, NI, DT, and WP performed field data collection and organized the database. TD and DT performed the statistical and economic analysis. TD wrote the first draft of the manuscript. TD, TR, DT, and WP provided the manuscript revision. All authors approved the submitted version.

## Funding

This study was technically supported by Mae On, San Kamphaeng, and Phatung Dairy Cooperatives, the Fifth Regional Livestock Office, and the Regional Field Epidemiology Training Program for Veterinarians, (R-FETPV), Department of Livestock Development (DLD), Thailand. Also, this study was made possible by the generous support of the American people through the United States Agency for International Development (USAID), under the Terms of Agreement Number OSRO/RAS/402/USA for immediate technical assistance to strengthen emergency preparedness for Highly Pathogenic Avian Influenza (HPAI) (Regional Activities).

## Conflict of interest

The authors declare that the research was conducted in the absence of any commercial or financial relationships that could be construed as a potential conflict of interest.

## Publisher's note

All claims expressed in this article are solely those of the authors and do not necessarily represent those of their affiliated organizations, or those of the publisher, the editors and the reviewers. Any product that may be evaluated in this article, or claim that may be made by its manufacturer, is not guaranteed or endorsed by the publisher.

## Author disclaimer

The contents of this article are the responsibility of the authors and do not necessarily reflect the views of USAID or the US Government.
