# Supplementary material for: An economic analysis of high milk somatic cell counts in dairy cattle in Chiang Mai, Thailand
Source: Front Vet Sci. 2022 Nov 4;9:958163. doi: 10.3389/fvets.2022.958163 (PMC9672387; doi:10.3389/fvets.2022.958163)
Supplement: Supplementary file 1 [file Data_Sheet_1.docx]

**Equations of Economic Analysis**

### Ex-post cost assessment of high SCC

*Ex-post cost assessment of high SCC at farm level for the 3-month period*

**Equation 1** Ex-post cost assessment of high SCC = Losses due to price reduction of milk due to high SCC + Discharged milk

$= \left[ \left( \left( P_{h} - P_{i July} \right)*31 \right)+ \left( \left( P_{h} - P_{i August} \right)*31 \right)+\left( \left( P_{h} - P_{i September} \right)*30 \right)* \mathrm{MilkS}_{\frac{kg}{day}} \right]+\left( \mathrm{Milk}_{\frac{\mathrm{diskg}}{\mathrm{day}}} * Ph*92 \right)$

**Equation 2** *Ex-post cost assessment of high SCC at the cluster level*

$$=\sum_{i=1}^{N} \left[ \left( \left( P_{h} - P_{i July} \right)*31 \right)+ \left( \left( P_{h} - P_{i August} \right)*31 \right)+\left( \left( P_{h} - P_{i September} \right)*30 \right)* \mathrm{MilkS}_{\frac{kg}{day}} \right]+\left( \mathrm{Milk}_{\frac{\mathrm{diskg}}{\mathrm{day}}} * Ph*92 \right)$$

To estimate the ex-post cost assessment of high SCC, we define the following variables:

*P_i July_ = Price of raw milk sold by farm i in July 2018*

*P_i_ _August_ = Price of raw milk sold by farm i in August 2018*

*P_i_ _September_ = Price of raw milk sold by farm i in September 2018*

*P_h_ = Regular price of raw milk (400,001 – 500,000 cells/ml) or 17.6 THB/kg*

*MilkS_kg/day_ = Number of kilograms of raw milk sold in average per day during the period*

*Milk _diskg/day_ = Number of kilograms of discharged milk in average per day during the period*

*N = number of farms in the cluster/cooperative*

### Estimation of Economic Losses of High SCC and cows with mastitis

Baseline scenario: Keeping a sick cow untreated and adding its milk into the farm’s milk tank

**Equation 3** Baseline profit = NPV (milk revenue) - NPV (Feed cost)

$$Baseline profit = \left[ \left( Qs*365 *Ps+ \left( \left( Nh-1 \right)*Qh*365*Ps \right) \right)+\left( \frac{Qs*365*Ps+\left( \left( Nh-1 \right)*Qh*365*Ps \right)}{1+DR} \right)+\left( \frac{Qs*365*Ps+\left( \left( Nh-1 \right)*Qh*365*Ps \right)}{\left( 1+DR \right)^{2}} \right) \right]- \left[ C*Nh+ \frac{C*Nh}{1+DR}+ \frac{C*Nh}{\left( 1+DR \right)^{2}} \right]$$

Strategy A: Culling a sick cow

In this case, farmers receive some revenue from selling the sick cow for slaughtering.

**Equation 4** Profit = NPV (milk revenue) + Selling revenue (sick cow) - NPV (Feed cost)

$$Profit= \left[ \left( \left( Nh-1 \right)*Qh*365*Ph \right)+\frac{\left( Nh-1 \right)*Qh*365*Ph}{1+DR} + \frac{\left( Nh-1 \right)*Qh*365*Ph}{\left( 1+DR \right)^{2}} \right]+Pcull- \left[ C*\left( Nh-1 \right)+ \frac{C*\left( Nh-1 \right)}{1+DR}+ \frac{C*\left( Nh-1 \right)}{{1+DR}^{2}} \right]$$

Strategy B.1: Successful treatment

A cow with mastitis is treated when famers can afford the treatment cost and expect that the cow will be fully recovered after the treatment. However, the milk during the period of treatment is prohibited to be sent to the cooperative due to the use of antibiotic and anti-inflammatory drug.

**Equation 5** Profit = NPV (milk revenue) - NPV (Feed cost) – Treatment costs

$$Profit= \left[ Qh*\left( 365-D \right)*Ph+\left( \left( Nh-1 \right)*Qh*365*Ph \right)+ \frac{Nh*Qh*365*Ph}{1+DR}+ \frac{Nh*Qh*365*Ph}{\left( 1+DR \right)^{2}} \right]- \left[ C*Nh + \frac{C*Nh}{1+DR}+ \frac{C*Nh}{\left( 1+DR \right)^{2}} \right]-T$$

Strategy B.2: Unsuccessful treatment followed by culling

In this case, after the treatment is unsuccessful, the famer sells the cow for slaughter, so the milk of the sick cow is never added to the milk tank

**Equation 6** Profit = NPV (milk revenue) + Selling revenue (sick cow) - NPV (Feed cost) – Treatment costs

$$Profit= \left[ \left( Nh-1 \right)*Qh*365*Ph+ \frac{\left( Nh-1 \right)*Qh*365*Ph}{1+DR}+\frac{\left( Nh-1 \right)*Qh*365*Ph}{\left( 1+DR \right)^{2}} \right]+Pcull- \left[ \left( \frac{C}{365}*D \right)+\left( C*\left( Nh-1 \right) \right)+ \frac{C*\left( Nh-1 \right)}{1+DR}+ \frac{C*\left( Nh-1 \right)}{\left( 1+DR \right)^{2}} \right]-T$$

To estimate the farm’s profit under the different scenarios, we define the following variables:

*Nh = Average number of healthy milking cows in a farm*

*Qh = milk produced by a healthy cow per day*

*Ps = average price penalized (sick cow)*

*Ph = Price of milk per kg (healthy cow)*

*Qs = milk produced by a sick cow per day*

*Pcull - Price of sick cow sold for slaughtering*

*C = annual feed costs*

*T = Treatment cost*

*D = Withdrawal period because of treatment*

*DR = Discount rate*
